# The EIPeptiDi tool: enhancing peptide discovery in ICAT-based LC MS/MS experiments

**DOI:** 10.1186/1471-2105-8-255

**Published:** 2007-07-15

**Authors:** Mario Cannataro, Giovanni Cuda, Marco Gaspari, Sergio Greco, Giuseppe Tradigo, Pierangelo Veltri

**Affiliations:** 1 Bioinformatics Laboratory, Experimental and Clinical Medicine Department, Magna Græcia University, viale Europa 88100, Catanzaro, Italy; 2 Proteomics Laboratory, Experimental and Clinical Medicine Department, Magna Græcia University, viale Europa 88100, Catanzaro, Italy; 3 Department of Electronics, Computer and System Sciences (DEIS), University of Calabria, via P. Bucci 41 C 87036, Rende, Italy

## Abstract

**Background:**

Isotope-coded affinity tags (ICAT) is a method for quantitative proteomics based on differential isotopic labeling, sample digestion and mass spectrometry (MS). The method allows the identification and relative quantification of proteins present in two samples and consists of the following phases. First, cysteine residues are either labeled using the ICAT Light or ICAT Heavy reagent (having identical chemical properties but different masses). Then, after whole sample digestion, the labeled peptides are captured selectively using the biotin tag contained in both ICAT reagents. Finally, the simplified peptide mixture is analyzed by nanoscale liquid chromatography-tandem mass spectrometry (LC-MS/MS). Nevertheless, the ICAT LC-MS/MS method still suffers from insufficient sample-to-sample reproducibility on peptide identification. In particular, the number and the type of peptides identified in different experiments can vary considerably and, thus, the statistical (comparative) analysis of sample sets is very challenging. Low information overlap at the peptide and, consequently, at the protein level, is very detrimental in situations where the number of samples to be analyzed is high.

**Results:**

We designed a method for improving the data processing and peptide identification in sample sets subjected to ICAT labeling and LC-MS/MS analysis, based on cross validating MS/MS results. Such a method has been implemented in a tool, called *EIPeptiDi*, which boosts the ICAT data analysis software improving peptide identification throughout the input data set. Heavy/Light (H/L) pairs quantified but not identified by the MS/MS routine, are assigned to peptide sequences identified in other samples, by using similarity criteria based on chromatographic retention time and Heavy/Light mass attributes. *EIPeptiDi *significantly improves the number of identified peptides per sample, proving that the proposed method has a considerable impact on the protein identification process and, consequently, on the amount of potentially critical information in clinical studies. The *EIPeptiDi *tool is available at  with a demo data set.

**Conclusion:**

*EIPeptiDi *significantly increases the number of peptides identified and quantified in analyzed samples, thus reducing the number of unassigned H/L pairs and allowing a better comparative analysis of sample data sets.

## Background

**Figure 1:**
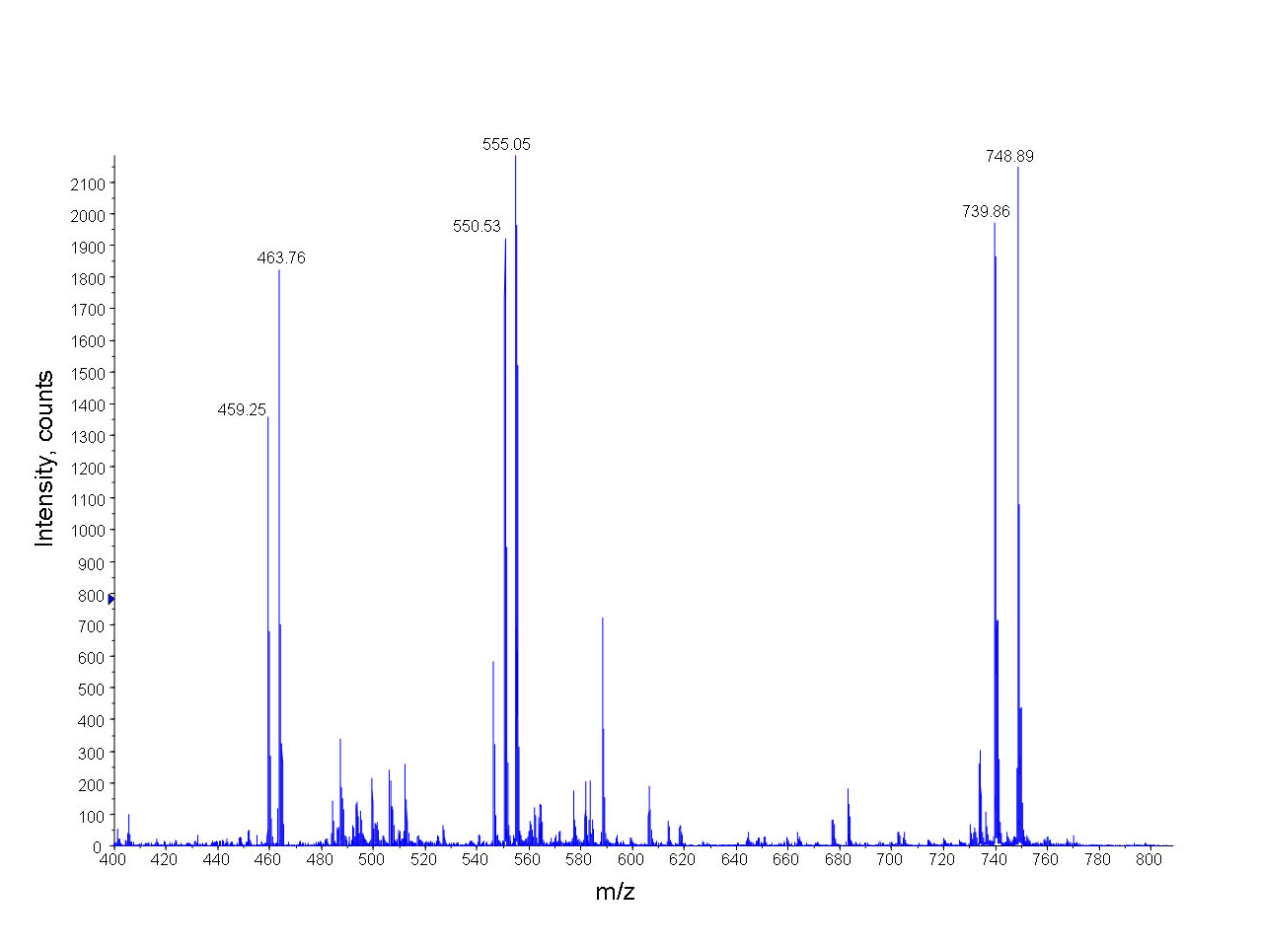
**Mass spectrum**. Mass spectrum of a biological sample (ICAT labeled peptide mixture).

Mass Spectrometry (MS) [1] is a powerful technique used to analyze biological samples, and it has been used to identify potentially important biomarkers in several human diseases. In short, it consists in associating a spectrum containing pairs of values [*m*/*z*, *intensity*] to the input biological sample [2]. Figure 1 shows an example of a MS spectrum where each [*m*/*z*, *intensity*] pair may be related to the presence of a biomolecule, e.g. a protein or portion of it (called peptide), present in the sample with mass to charge ratio *m*/*z *and abundance expressed by the *intensity *value [3,4].

Currently, there exist many instruments and techniques for generating spectra from biological samples as well as many software platforms for managing experiments and identifying proteins contained in the original samples. An MS-based methodology which is being extensively applied in biological research is the shotgun LC-MS/MS approach. It consists of three main steps: i) enzymatic digestion of a protein mixture; ii) separation of generated peptides through single or multiple steps of chromatographic separation; iii) MS analysis through tandem mass spectrometry (MS/MS). Enzymatic digestion activity breaks down the starting proteins in small portions (peptides), which can be more efficiently separated by chromatography. Furthermore, peptides are much more suitable for MS/MS sequencing than their corresponding intact proteins.

The MS/MS process consists in performing multiple steps of mass spectrometric analysis by generating a mass spectrum of the fragments derived from a selected peptide peak isolated in a previous MS stage. The fragments, produced via breakdown of the parent peptide through gas collisions, can be correlated to amino acid sequences by dedicated search programs [5]. Protein/peptide identification from MS/MS spectra consists in the computation of *qualitative *information and is performed by querying publicly available databases (e.g. the *SwissProt *database [6] queried using *Mascot *[7]). Proteomics literature presents an excessive fragmentation of repositories and tools used for storing and handling large scale MS/MS protemoics results. In order to meet requirements for more systematic analysis and representation of proteomics data, the Proteomics Standards Initiative (PSI) [8] has been created by the Human Proteome Organisation (HUPO) with the aim of defining community standards and, thus, facilitating data exchange and public availability of data.

Increasing attention has also been devoted to fully exploiting the *quantitative *information, such as protein abundance in complex mixtures, obtained by LC-MS/MS experiments [9-11]. Recently developed tools, such as *MSight *[12] and *Pep3D *[13], transform LC-MS full scan data into two-dimensional (2D) images and then manage them using 2D gel electrophoresis analysis techniques. Other tools, such as *msInspect *[14], *LCMS-2D *[15] and *MZmine *[10,16], locate peptide signals within LC-MS data, calculate signal intensities/peak areas and compare multiple data files. All these tools provide a graphical interface for data visualization and analysis.

As regards the quantitative aspects, the simple detection of the ion intensity of peptide peaks in MS is not usually an accurate way of acquiring information about its abundance. MS quantification can be improved by using isotopic labeling methods [17] which allow to measure the relative abundance of Heavy-labeled peptides with respect to Light-labeled peptides of a reference sample. Isotope-coded affinity tags (ICAT) [18] is currently one of the most widely adopted isotopic labeling approaches.

**Figure 2:**
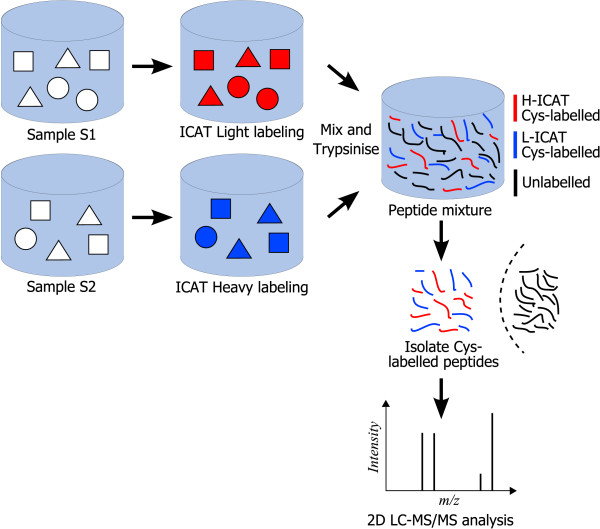
**ICAT protocol**. Schematic representation of the ICAT protocol.

The ICAT protocol, reported in Figure 2, consists in marking two protein mixtures (sample S1 and sample S2) with, respectively, Heavy (H) and Light (L) labels having identical chemical properties but different masses. The ICAT label marks all cysteines present in the samples by relying on a thiol-reacting group. After mixing the two samples (S1 and S2) and performing enzymatic digestion, the ICAT-labeled peptides are selectively captured by affinity chromatography using the biotin tag present in the ICAT reagent. LC-MS/MS analysis of the purified peptide mixture (peptides containing cysteine) allows the detection of hundreds to thousands of peak *pairs *corresponding to peptides marked with either label L or label H. Identical peptides belonging to the same protein, but originating in different samples (either sample *S*_1 _or *S*_2_) are detected at different *m*/*z *values because of the difference in mass between the L and the H reagents. For instance, in Figure 1 the peak pairs (463.76, 459.25), (555.05, 550.53) and (748.89, 739.86), where the first two pairs are doubly charged ions, whereas the third one is singly charged, correspond to H/L pairs and they have delta masses equal to 9.02 (= (463.76 - 459.25) × 2), 9.04 (= (555.05 - 550.53) × 2) and 9.03 (= 748.89 - 739.86) Da, respectively. The ratio of MS intensities between the H and L forms within a peak *pair *(H/L ratio) provides accurate relative quantitative information on the abundance of a particular peptide, and thus the corresponding protein, in sample S2 with respect to its abundance in sample S1. In ICAT-based experiments, LC-MS/MS analysis is normally performed in data-dependent mode. This means that, during the chromatographic separation of peptides, the mass spectrometer automatically switches from full scan *MS mode*, which allows the detection of H/L pairs at a particular chromatographic retention time *t*, to *MS*/*MS mode *on the most abundant peaks (typically 2–5 peaks) present in the MS spectrum at time *t*.

**Figure 3:**
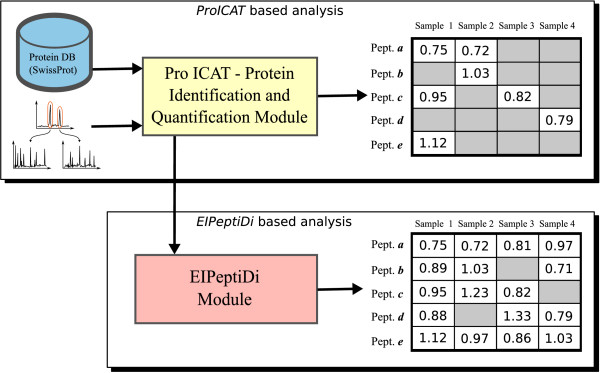
**Peptide discovery**. The *ProICAT *and *EIPeptiDi *protein identification processes.

After database search, *qualitative *information (peptide sequence identification *via *MS/MS) is correlated to *quantitative *information (H/L ratios) in order to produce tables of proteins/peptides (quality sample contents) with their relative expression levels (quantity sample contents). Figure 3 shows the protein/peptide identification process performed using the Applied Biosystems (AB) *ProICAT *module [19] which is in charge of identifying proteins/peptides by querying a protein database. Furthermore, *ProICAT *generates a list of H/L pairs by treating the full scan information of the LC-MS/MS data as an intensity image and then detecsting chemical species through the 3D LCMS Reconstruct algorithm present in the BioAnalyst software. For each isotope series, the algorithm checks for the other isotope series separated by the neutral mass difference of the two forms of the ICAT reagent.

**Figure 4:**
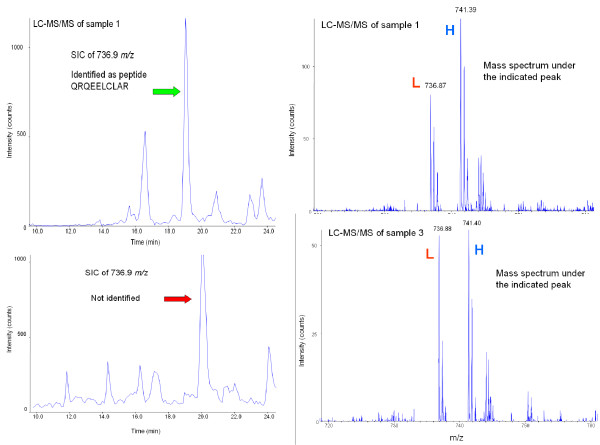
**Example of missing value**. Selected Ion Chromatograms (SICs) illustrate how an H/L pair having the same *m*/*z *and retention time values of peptide QRQEELCLAR, identified in Sample 1 is also present in the LC-MS/MS data of Sample 3.

The table shown on the upper, right of Figure 3 depicts a simplified example of a *ProICAT *result, where the rows denote peptides, columns denote samples and each entry value corresponds to an *H*/*L *ratio (quantitative information). A significant disadvantage of the ICAT LC-MS/MS protocol is that the number of identified peptides varies from experiment to experiment (see missing values in the upper right table of Figure 3), making the statistical analysis of sample sets very challenging. Experimental observations showed us that, at least in the case of plasma/serum samples, the missing values are almost always caused by the variability of the peptide identification process rather than by the absence of a particular protein in a given sample. Indeed, in experiments performed on different samples we noted that expected peptides were not always identified by the *ProICAT *routine. In a 7 sample human serum data set (denoted by Sample 1, ..., Sample 7), the peptide QRQEELCLAR, belonging to plasma retinol-binding protein, was identified in only two of the seven samples by *ProICAT *(see Table 1), while the protein was expected to be present in all samples and its presence was also confirmed by manual inspection of LC-MS/MS full scan raw data. Figure 4 shows Selected Ion Chromatograms (SICs) for the L labelled QRQEELCLAR peptide identified in Sample 1 and the corresponding SIC obtained from Sample 3. The H/L pair present in the LC-MS/MS data of Sample 3, having the same *m*/*z *values and retention time as peptide QRQEELCLAR, is strongly suspected of corresponding to the same peptide identified in Sample 1. In our experience, proteins detected by ICAT LC-MS/MS analyses were, in all cases, already known to be present in blood plasma/serum. For some of these proteins, laboratory reference values are also available [20], whereas other proteins have been less investigated, but nevertheless have been identified in previous studies on serum/plasma proteome [21]. All these observations confirmed that, concerning ICAT-based LC-MS/MS plasma/serum analyses, missing values are mostly due to variability in the MS/MS identification process. The main weakness in current ICAT-based proteomics platforms, when dealing with a considerable number of samples, lies in the insufficient overlap of information between the different samples. Moulder *et al*. [22] have compared some ICAT data analysis software and have shown that *ProICAT*, *Spectrum Mill *and *SEQUEST *give comparable results in terms of protein quantification, but different, and in some cases complementary, results in terms of protein identification. Nevertheless, none of these three data analyses softwares have proposed a solution to improve data overlap. Cross-talk between LC-MS/MS data has not been applied to data generated after isotopic labeling, even though the concept of cross-talk has already been introduced in [23] and [24]. The systematic evaluation of qualitative and quantitative information of LC-MS/MS data in multiple experiments was addressed as an open topic in a recent bioinformatics review [25]. Indeed, recent works on LC-MS data analysis do not make use of the precious qualitative information given by MS/MS spectra [10,26]. In particular, the importance of merging MS/MS identifications when a high number of samples is analyzed, has been underestimated and never applied to the ICAT pipeline process or to any other LC-MS/MS-based quantitative proteomics approach (e.g., Stable isotope labeling with amino acids in cell culture, *SILAC *[27]). The technique proposed here fills this gap and its implementation is freely available on line.

**Table 1:** Example of ICAT results. Real-life example of an ICAT results table; seven serum samples (*labelled H*) were analysed against a reference serum sample (*labelled L*).

**Protein name**	**ICAT peptide sequence**	**H/L Sample 1**	**H/L Sample 2**	**H/L Sample 3**	**H/L Sample 4**	**H/L Sample 5**	**H/L Sample 6**	**H/L Sample 7**
Alpha-2-macroglobulin	VTAAPQSVCALR	0.46	0.56	1.02	0.38	0.44	0.90	0.82
Alpha-1-antitrypsin	LGMFNIQHCK	1.11	1.82	2.01	1.01	0.98	0.93	1.54
Alpha-2-HS-glycoprotein	EHAVEGDCDFQLLK	1.16	-	1.07	0.82	0.98	1.05	0.78
Alpha-1-acid glycoprotein 2	EQLGEFYEALDCLR	1.12	1.96	0.97	0.99	-	1.81	-
AMBP protein precursor	TVAACNLPIVR	1.12	1.22	1.02	-	1.44	1.21	1.48
Apolipoprotein A-II	EPCVESLVSQYFQTVTDYGK	1.61	-	1.21	1.04	1.08	0.92	1.32
Vitamin D-binding protein	HQPQEFPTYVEPTNDEICEAFR	-	1.45	1.23	1.14	1.06	0.94	1.12
Plasma retinol-binding protein	QRQEELCLAR	1.68	1.55	-	-	-	-	-

## Implementation

In this paper we present a technique, implemented in a tool called *EIPeptiDi *(for *Enhanced ICAT Peptide Discovery*), that improves protein identification in ICAT based experiments. The main module is based on a cross validation algorithm that tries to associate Heavy (H) or Light (L) peaks, *quantified *by the *ProICAT *software [19], but not assigned by the MS/MS routine and thus *not identified*, to peptide sequences identified in other experiments of the same sample set.

*EIPeptiDi *is composed of the following main modules: (i) the database wrapper, (ii) the data calibration module, (iii) the cross validation module and (iv) the graphical user interface (GUI). Starting from the *ProICAT *results, the database wrapper extracts data consisting of peak measures, which may be (or may not be) assigned to peptides. The data calibration module is in charge of aligning chromatographic retention time information to improve the cross validation phase. The cross validation module allows to increase the number of peak measures assigned to peptides, and, consequently, to increase the number of identified proteins. Finally, the GUI, based on Java web start technology [28], allows *EIPeptiDi *to be run in a web browser. In the following the structure of the source data and the algorithms used by the main modules of *EIPeptiDi *are described. To facilitate the understanding of the protein identification boosting method, the cross validation algorithm is described before the calibration one.

### The cross validation algorithm

The *ProICAT *software produces a Microsoft Access database instance containing information about the performed experiments. In particular, the database contains information about peak measures, identified peptides and proteins, samples, instruments used and their setting parameters, and others. The role of the wrapper is to extract information which are useful for the next tasks. More specifically, the wrapper builds a new "integrated" database containing information about

• proteins, e.g. protein name and species;

• peptides, e.g. peptide amino acid sequence;

• samples, e.g. sample identifier, description, date in which the analysis has been performed;

• ICAT measures, e.g. mass, measure type (H or L), starting and ending chromatographic times;

• associations between ICAT measures and peptides, ICAT measures and samples, and peptides and proteins.

Using this information *ProICAT *computes, for each sample, a list of measures which can be associated to peptides and proteins. Upper right part of Figure 3 shows a simplification of the output where only the *H*/*L *ratio of assigned peptides to samples is reported. Nevertheless, *ProICAT *result contains many quantified peaks that are not associated to identified peptides. Indeed, by using *ProICAT *we observed that the number of quantified peaks from a LC-MS/MS run on one biological sample is typically much higher than the number of peptides identified, meaning that many quantified peaks have not been assigned to any peptide (see missing values in Table 1). According to [14] the output of an ICAT-based LC-MS/MS experiment contains thousands of quantified peak pairs. Nevertheless, by performing several experiments, we observed that, usually, only few hundreds of them can be successfully identified. Moreover, running multiple experiments on the same sample, we noted that the overall number of identified peptides increases, meaning that each LC-MS/MS result contains many more features than what can be identified by the MS/MS routine. Thus, it is feasible to design a framework that increases the number of identified peptides by *comparing *qualitative and quantitative information of multiple LC-MS/MS results.

**Figure 5:**
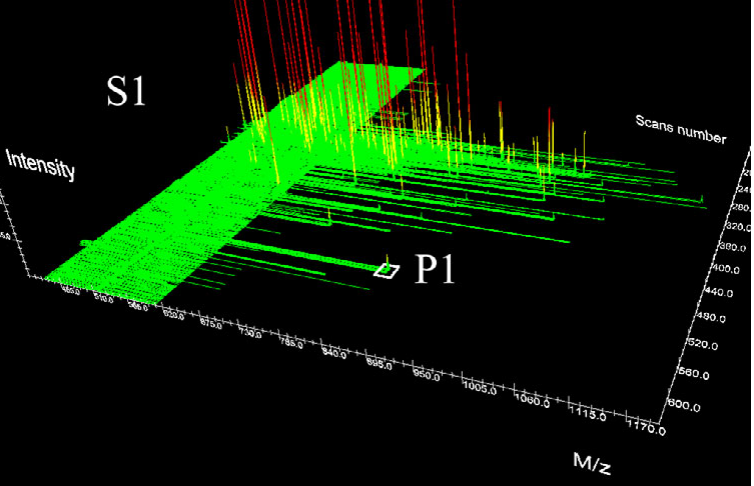
**MS/MS Sample**. In Sample S1 the peptide P1 is identified.

**Figure 6:**
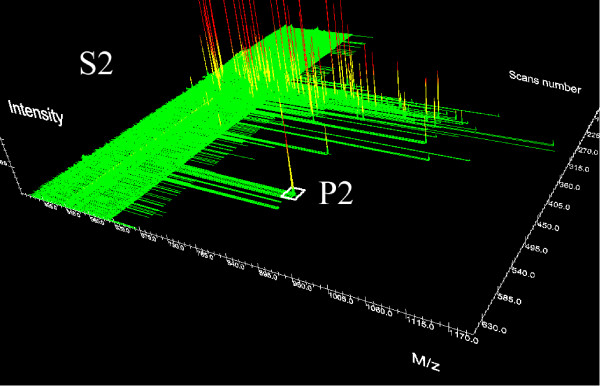
**Peptide results comparison**. Comparing MS/MS results on two samples: in Sample S2, the peptide P2 is not identified through MS/MS database search. Nevertheless, it can be identified via (retention time, *m*/*z*) matching with peptide P1 in Sample S1 (see Figure 5).

In order to assign identified peptides to quantified peaks, the similarity of peaks belonging to different samples is computed. The similarity measure is based on the comparison of mass values and chromatographic retention times which characterize uniquely peaks. For instance, let us consider the LC-MS/MS data shown in Figures 5 and 6 (only full scan information is displayed) and assume that peak P1, detected in the LC-MS/MS run of sample *S*_1_, is successfully identified by MS/MS, whereas in sample *S*_2 _the peak P2 is detected (but not identified) at the same *m*/*z*, *retention time *as the peak P1. Then, we can assign the same peptide sequence of P1 to the peak P2. Since peak matching has to take into account experimental errors, appropriate tolerance intervals have to be defined for both *m*/*z *and retention time. We call such intervals *mass tolerance *and *retention time tolerance*. Peak P2 in Figure 6 is thus assigned to the same peptide sequence of P1, if its *m*/*z *and retention times are equal to the *m*/*z *and retention time values of P1 within an error defined by the two tolerance values.

The accuracy of the method varies with the definition of such tolerance values. Large tolerance windows may lead to false hits. In our initial tests we used a delta retention time tolerance between 3 and 5 minutes and a mass tolerance of 0.003% (30 parts per million). Experiments have shown that such values considerably reduce the risk of false hits, while maximizing the newly detected proteins/peptides (see Section *EiPeptiDi tolerance value evaluation*). In the following we sketch the identification algorithm implemented in *EIPeptiDi *to boost the *ProICAT *peptide identification, by exploiting the experimental observations reported above.

Let *F *be the set of identified (found) peptides in all samples. *F *is the set of tuples *t *= (*p*, *St*, *Et*, *m*, *mty*, *S*_*id*_) where *p *is the peptide name detected (found) in the sample *S*_*id *_at retention time interval (*St*, *Et*), where *St *stands for start time and *Et *for end time, and at mass (*m*, *mty*) where *m *stands for the mass value and *mty *may assumes respectively *Heavy *or *Light *value. Analogously, *NF *is the set of (*not found*) tuples *t *= (⊥, *St*, *Et*, *m*, *mty*, *S*_*id*_) of measured peaks, i.e. masses and retention times measures, in the sample *S*_*id *_which are not associated with any peptide (the null value ⊥ states that the measure is not assigned to any peptide). Moreover, given a tuple *t *belonging to either *F *or *NF*, the notation *t*[*a*_*i*_, ..., *a*_*k*_] denotes the projection of *t *over the attributes *a*_*i*_, ..., *a*_*k*_. In the following we present a simplified version of the algorithm.

**procedure **Peptides_Discovery(*F*, *NF*)

// *F *contains the peptides found

// *NF *contains masses and retention times not assigned to any peptide

**const ***MAX_ MT *= 0.00003; // mass tolerance 30 ppm

**const ***MAX_ RTT *= 3; // retention time tolerance in minutes

**const ***minSup *= 0.75; // minimum support to assign not found measures

**var **Δ*m*, Δ*St*, Δ*Et*: **real**;

begin

   **for **i = 1 **to **|*NF*| **do begin**

      // for all tuples in *NF *try to assign a peptide

      *TMP*_*i *_= ∅;

      // *TMP*_*i *_is a multiset containing temporarily assigned peptides

      **for **j = 1 **to **|*F*| **do begin**

         //search in all tuples in *F*

         //calculate mass tolerance for *t*_*i*_[*m*]

         Δ*m *:= MAX_ MT * *t*_*j*_[*m*];

         Δ*St *:= **abs**(*t*_*i*_[*St*] - *t*_*j*_[*St*]);

         Δ*Et *:= **abs**(*t*_*i*_[*Et*] - *t*_*j*_[*Et*]);

         // Verify mass and retention time falls in Δ*time *intervals.

         // and that both masses are Heavy or Light

         **if **((*t*_*j*_[*m*] - Δ*m *<*t*_*i*_[*m*] <*t*_*j*_[*m*] + Δ*m*) **and ***t*_*i*_[*mty*] = *t*_*j*_[*mty*] **and**

            Δ*St *≤ *MAX_ RTT*/2 **and **Δ*Et *≤ *MAX_ RTT*/2) **then begin**

               // Assign (temporarily) the peptide *t*_*j*_[*p*] to *t*_*i*_

               *TMP*_*i *_= *TMP*_*i *_∪ {*t*_*j*_[*p*].*t*_*i*_[*St*, *Et*, *m*, *mty*, *S*_*id*_]};

               *NF *= *NF *- {*t*_*i*_};

         **end**;

      **end**;

         **if **∃ peptide p^
 MathType@MTEF@5@5@+=feaafiart1ev1aaatCvAUfKttLearuWrP9MDH5MBPbIqV92AaeXatLxBI9gBaebbnrfifHhDYfgasaacH8akY=wiFfYdH8Gipec8Eeeu0xXdbba9frFj0=OqFfea0dXdd9vqai=hGuQ8kuc9pgc9s8qqaq=dirpe0xb9q8qiLsFr0=vr0=vr0dc8meaabaqaciaacaGaaeqabaqabeGadaaakeaacuWGWbaCgaqcaaaa@2E25@ s.t. |*t*|*t *∈ *TMP*_*i *_∧ *t*[*p*] = p^
 MathType@MTEF@5@5@+=feaafiart1ev1aaatCvAUfKttLearuWrP9MDH5MBPbIqV92AaeXatLxBI9gBaebbnrfifHhDYfgasaacH8akY=wiFfYdH8Gipec8Eeeu0xXdbba9frFj0=OqFfea0dXdd9vqai=hGuQ8kuc9pgc9s8qqaq=dirpe0xb9q8qiLsFr0=vr0=vr0dc8meaabaqaciaacaGaaeqabaqabeGadaaakeaacuWGWbaCgaqcaaaa@2E25@}| > |*TMP*_*i*_| × *minSup ***then**

            *F *= *F *∪ {p^
 MathType@MTEF@5@5@+=feaafiart1ev1aaatCvAUfKttLearuWrP9MDH5MBPbIqV92AaeXatLxBI9gBaebbnrfifHhDYfgasaacH8akY=wiFfYdH8Gipec8Eeeu0xXdbba9frFj0=OqFfea0dXdd9vqai=hGuQ8kuc9pgc9s8qqaq=dirpe0xb9q8qiLsFr0=vr0=vr0dc8meaabaqaciaacaGaaeqabaqabeGadaaakeaacuWGWbaCgaqcaaaa@2E25@.*t*_*i*_[*St*, *Et*, *m*, *mty*, *S*_*id*_]};

   **end**;

   **Return ***F*, *NF*, ∪_*i *= 1...|*NF*|_*TMP*_*i*_;

**end **Peptides_Discovery;

The constants *MAX_ MT *and *MAX_ RTT *represent the mass and retention time tolerances, whereas *minSup *is a constant whose value is contained in the interval [0..1] and defines the minimum threshold to assign a peptide to a not found measure. Such parameters may be defined by the user (via a dialog box), taking into account the MS instrument resolution and chromatographic performance. In our experiments we used, respectively, *MAX_ MT *= 30 *ppm *and *MAX_ RTT *= 3 *minutes*. Such parameters have been validated by several experiments on the *EIPeptiDi *tool. Moreover, the tolerance parameters may be optimized if input spectra are *calibrated*, with respect to retention time and mass values. As input spectra produced by MS instruments are already calibrated with respect to mass values, in the next section we present the algorithm implemented in *EIPeptiDi *performing the calibration of spectra with respect to retention time.

### Data calibration

*EIPeptiDi *implements a simple retention time calibration module based on a linear interpolation algorithm. The basic idea consists in considering the set of peptides found in *all *samples and selecting a small subset (e.g. 10 measures) chosen across the whole chromatographic time interval, that are used for evaluating interpolated lines. The calibration is performed with respect to a selected input sample, e.g. *S*_1_, that becomes the reference sample for realigning chromatographic time of the remaining samples. Let *N *be the number of samples, and let *M *be the number of selected peptides found in all samples. The algorithm consists in evaluating *N *- 1 interpolated lines of equation *f*_*i*_(*x*) : *y *= *α*_*i*_*x *+ *β*_*i *_for each sample *S*_*i *_(*i *= 2..*N*), where the *x *axis represents the reference chromatographic time for the sample *S*_1 _and the *y *axis represents the chromatographic time for the sample *S*_*i *_that must be calibrated. The *α*_*i *_and *β*_*i *_coefficients of the *i*_*th *_linear equation are evaluated by interpolating the retention times of the *M *peptides respectively for the samples *S*_1 _and *S*_*i*_. Then, the chromatographic retention time information relative to all the quantified (but not identified) peptides in the sample *S*_*i *_are recalculated according to the calibration linear function.

**Figure 7:**
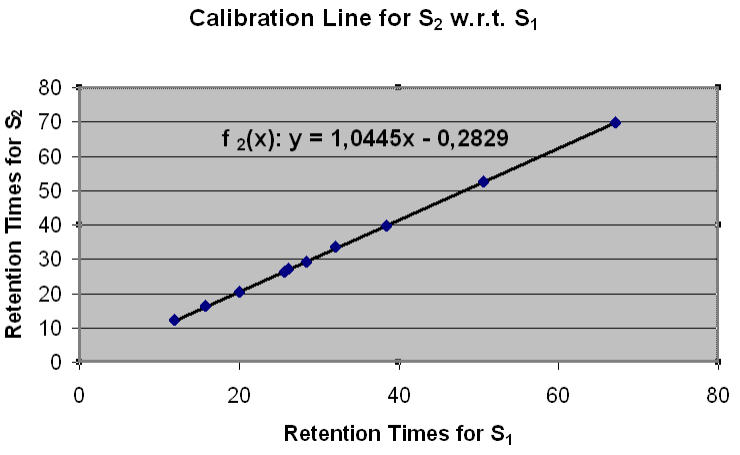
**Retention time calibration by linear interpolation**. The interpolation line used to calibrate retention time in Sample *S*_2 _with respect to *S*_1_.

For instance, let us consider an experiment performed on N = 7 samples, denoted by *S*_1 _... *S*_*N*_, and let *S*_1 _be the reference sample; let *p*_1_, ..., *p*_*M*_, with *M *= 10, be the reference peptides quantified and identified in all N samples. The calibration algorithm performs in N-1 iterations evaluating N-1 calibration linear equations. Table 2 reports data used to calibrate the sample *S*_2 _with respect to *S*_1_. The first column contains the amino acid sequences of the selected common peptides, called *landmark peaks*; the second and third columns contain retention times of landmark peaks found in *S*_1 _and *S*_2_. Such times differ on average by 3.33%. The calibration linear equation is the following *f*_2_(*x*) : *y *= 1.0445*x *- 0.2829 (see Figure 7). Such an equation is used to calibrate retention times for *all *Heavy/Light peak pairs in sample *S*_2_. For instance, the calibrated retention time for the DYFMPCPGR peptide is now 28.39 minutes, which is very close to the retention time of DYFMPCPGR in *S*_1 _(28.36 minutes), whereas the retention time before calibration was 29.28. The average difference among the M landmark peaks is now reduced to 0.56%.

In the following we present the calibration algorithm implemented in *EIPeptiDi*.

**Table 2:** Retention times used for data calibration. Retention times of landmark peaks used to calibrate sample *S*_2 _with respect to reference sample *S*_1_.

**Peptide sequences**	**Retention Times in *S*_1_**	**Retention Times in *S*_2_**
VANPCVK	11.87	12.22
WCALSHHER	15.76	16.21
KPVDEYKDCHLAQVPSHTVVAR	19.99	20.42
FSGQLNSHGCFYQQVK	25.68	26.32
CLVEKGDVAFVK	26.10	27.06
DYFMPCPGR	28.37	29.28
GPSVFPLAPCSR	32.01	33.48
KGDTFSCMVGHEALPLAFTQK	38.43	39.75
DLYSGLIGPLIVCR	50.59	52.61
EPCVESLVSQYFQTVTDYGKDLMEK	67.07	69.74

**procedure **LinearDataCalibration(*F*, *NF*, *S*)

// F contains the peptides found within samples with masses, retention times

// NF contains masses and retention times not assigned within samples

// Let S = {*S*_1_, ..., *S*_*N*_} be the set of samples

**const ***NB_PEPT *= 10; //number of points (peptides) for calibration

begin

   //Select NB_ PEPT peptides among the set of found F

   PEPT_SET = **SelectPeptides**(F, NB_PEPT) identified in all samples

   **for ***i *= 2 **to *N *do begin**

      //evaluate the interpolation line *f*_*i*_(*x*) = *α*_*i*_*x *+ *β*_*i*_;

      *f*_*i*_(*x*) = EvaluateLinearInterpolation(*S*_1_, *S*_*i*_, PEPT_SET);

      //calibrate all retention times of all Heavy-Light pairs in *S*_*i*_

      *S'*_*i *_= Calibrate(*f*_*i*_(*x*), *S*_*i*_);

      **Return {***S*_1_, *S'*_*i*_, ..., *S'*_*N*_**}**;

   **end**;

**end **Linear DataCalibration;

Even if there exist several proposals for chromatographic time realignment of LC-MS data based on landmark peaks, [29-31], we used a linear calibration function which has given good results and allows to validate results in a simple way. Moreover, as data calibration is an independent task, more sophisticated alignment strategies could be used.

**Figure 8:**
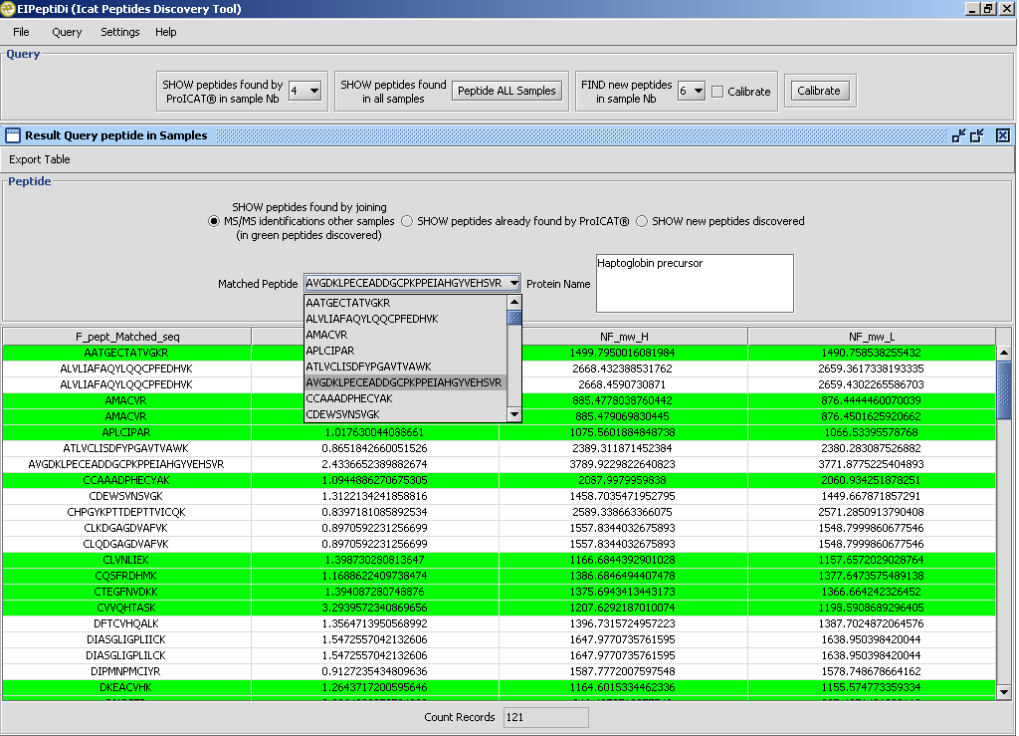
**Discovered peptides by using EIPeptiDi tool**. *Discovered *peptides are highlighted.

Logical functionalities described above have been fully implemented in the *EIPeptiDi *tool using the Java programming language. Figure 3 shows how the *EIPeptiDi *tool fits in the MS/MS data enhancement process. It takes in input *ProICAT *results and enriches them with additional identified peptides (see table in the lower, right side of Figure 3). Figure 8 reports the graphical user interface of an *EIPeptiDi *execution, where the highlighted rows represent the discovered peptides associated to biological input samples. Users may define the *Delta RT *and the *Delta mass *tolerances using expected chromatographic reproducibility and instrument mass accuracy.

## Results

This section presents some of the performed experiments. Firstly, used data sets are described, then parameters setting is presented and, finally, experimental results are reported.

### Data sets description and preparation

*EIPeptiDi *has been tested on two data sets containing seven and ten collection of LC-MS/MS generated samples (denoted, respectively as data set A and data set B). A third data set has been made available on-line for testing. In all cases, samples were human sera subjected to albumin/IgG depletion, ICAT-labeling and tryptic digestion before LC-MS/MS analysis. Concerning the immunodepletion step, it is a widely accepted approach to remove highly abundant proteins from serum before proteomic analysis. This step may contribute to increase the experimental error and it might also cause a specific loss of some proteins [32]. Nevertheless, the increase of dynamic range obtained by such a procedure dramatically improves proteome coverage in serum, as demonstrated by [33]. Furthermore, removal of high abundance proteins is highly recommended [34], in cases where the analytical strategy is based on enrichment of cysteine containing peptides.

The two data sets A and B contain serum samples kindly provided by clinical colleagues of University Magna Graecia Medical School. In both data sets, Heavy (H) labeled samples were generated either from healthy individuals or diseased patients; they all were compared with a reference, Light (L) labeled sample. In the following, sample preparation and analysis is described.

Blood samples were collected after informed consent. Approximately 8 ml of blood was drawn by venipuncture and placed on ice. The samples were centrifuged within 2 hours of collection at 1.400 × g for 10 minutes, and serum was aliquoted into Nalgene tubes and stored at -80°*C*. Sera were depleted of albumin and immunoglobulins by using ProteoExtractTM HSA/IgG (human serum albumin/immunoglobin G) Removal Kit (Calbiochem). Albumin and IgG-depleted serum fractions were precipitated at -20°*C *with cold-acetone in 1:7 v/v ratios. The protein pellet was then dissolved in 50 mM Tris and 0,1% SDS buffer pH 8.5, labeled with the Cleavable ICAT Reagent Kit for protein Labeling [19] (either H or L), digested and purified according to manufacturer's instructions.

Chromatography was performed on an Ultimate nano LC system from Dionex [35]. All chromatographic columns used were also from Dionex. The ICAT-labelled peptide mixture was dissolved in 200 *μL *of loading pump solvent, consisting of water/acetonitrile/trifluoroacetic acid (TFA) 98/2/0.1 (v/v/v). 10 *μL *of the peptide solution were then injected for LC-MS analysis. Peptides were loaded onto a 0.3 × 5 mm Pepmap C18 trapping column, using the loading solvent at constant flow rate of 30 *μL*/*min*, and subsequently eluted through an analytical nanoLC column, 0.075 × 150 mm, packed with Pepman C18 3 *μm *silica particles. For gradient elution of peptides, mobile phase A was water/acetonitrile/formic acid (FA)/TFA 97.9:2:0.08:0.02 (v/v/v/v) and mobile phase B was water/acetonitrile/FA/TFA 4.9:95:0.08:0.02 (v/v/v/v). Gradient was from 5 to 45% B in 80 minutes at 300 nL/min flow rate.

MS detection was performed on a QSTAR XL hybrid LC-MS/MS from Applied Biosystems [19] operating in positive ion mode, with nanoelectrospray potential at 1800 V, curtain gas at 15 units, CAD gas at 3 units. Information-dependent acquisition (IDA) was performed by selecting the two most abundant peaks for MS/MS analysis after a full TOF-MS scan from 400 to 1600 *m*/*z *lasting 2 seconds. Both MS/MS analyses were performed in enhanced mode (2 seconds/scan). Threshold value for peak selection for MS/MS was 20 counts. Qualitative and quantitative LC-MS/MS information was processed by the *ProICAT *software. The Swiss Prot database was queried for protein identification using the following settings: peptide mass tolerance at 0.05 Da; MS/MS tolerance at 0.5 Da; mod. tolerance 1 Da; confidence level greater than 95%.

### EIPeptiDi tolerance value evaluation

In order to assess the best tolerance for mass and retention time values in a systematic way, we performed experiments on data sets A and B. For each distinct data set, the subset of peptides found in all samples was selected (43 peptides for data set A and 34 peptides for data set B). Then, for both data sets, the first sample was taken as reference. For all remaining samples in each data set, and for each selected peptide, the differences in mass and retention time values with respect to the mass and retention time of the corresponding peptide in the reference sample (of the data set) were calculated.

The average difference between mass values of peptides, equal to 7 ppm (parts per million) has been calculated for both data sets A and B. The standard deviation on this measurement was 6 ppm, while the maximum difference observed was 25 ppm for both data sets. Considering that the subsets under consideration represented high quality data (i.e. high intensity peaks denoting a better mass accuracy than the rest of the mass measurements in the data sets), we chose a value of 30 ppm as default mass tolerance. As regards retention time, results confirmed the importance of the calibration step performed as discussed in Section *Data calibration*. Results are summarized in Table 3 where the values obtained concerning maximum difference and average difference (*plus *its associated standard deviation), indicated that the optimal retention time tolerance to be used after chromatographic time alignment was in the range 0.7–1.5 min. Instead, not-calibrated data would have required much higher tolerance values (3–4 min). We chose a tolerance of 1.5 minutes for subsequent experiments, also taking into account the compromise between the number of new peptides found and the rate of false positive.

**Table 3:** Retention time differences. Maximum and average retention time differences between peaks of reference peptides (sample S1) and the corresponding peaks of peptides in samples S2 to S7 in data set A (subset of peptides found in all samples).

	**Data set A**	**Data set A recalibrated**	**Data set B**	**Data set B recalibrated**
**Maximum RT difference**	3.36	1.66	3.68	1.28
**Average RT difference**	1.24	0.67	1.80	0.32
**Standard deviation**	0.31	0.19	1.29	0.15

The tolerance values found for data sets A and B prove that it is possible to calculate such values reliably by using the subsets of peptides found in all samples of the data set itself.

### EIPeptiDi on data sets A and B

**Figure 9:**
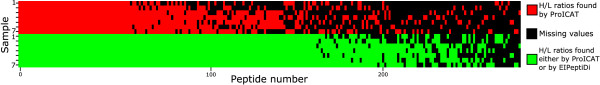
**Result data matrix**. *Missing values *decrease using *EIPeptiDi*. Each column represents a different peptide sequence, while each row represents a sample. Colored boxes indicate that a H/L ratio is available for the corresponding peptide.

**Figure 10:**
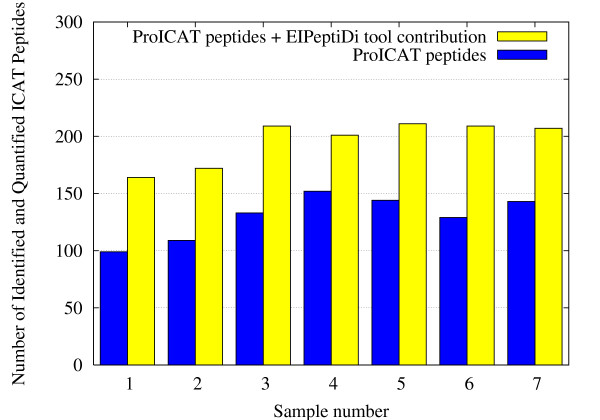
**Data set results**. Number of identified and quantified peptides per sample with and without the use of *EIPeptiDi *in the 7 samples data set A.

**Figure 11:**
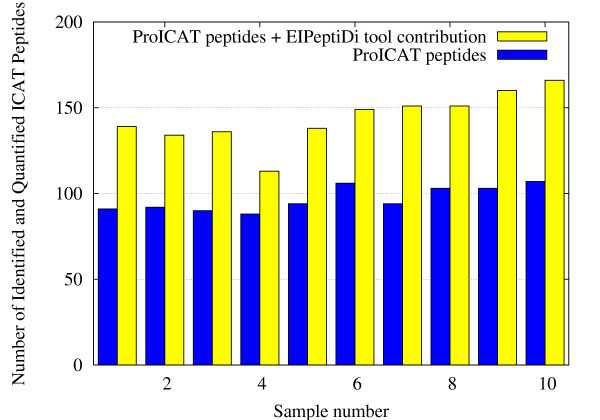
**Data set results**. Number of identified and quantified peptides per sample with and without the use of *EIPeptiDi *in the 10 samples data set B.

The improvements in data analysis can be appreciated in Figure 9, where the whole matrix of peptides found in data set A is schematized. Black colored rectangles indicate missing values. The top part of the Figure shows the peptides identified by the *ProICAT *procedure, while the bottom one shows those identified by *EIPeptiDi*. The bottom part of Figure 9 shows a significant decrease in the occurrence of missing values, where peptides having their associated H/L ratio are indicated as green rectangles (gray for black and white printed paper). Moreover, the number of peptides identified and quantified in *all *the 7 samples (full colored in Figure 9), increased dramatically using *EIPeptiDi*. Considering the experimental results without *EIPeptiDi*, 53 identified and quantified peptides were common to all samples, belonging to 19 distinct proteins. Using *EIPeptiDi*, this number raised to 139 peptides corresponding to 40 distinct proteins. This performance boost is also shown in Figures 10 and 11 that report the increment in the number of identified and quantified peptides *per sample *for the data set A and B. For data set A, the average number of identified peptides per sample raised from 129 to 196. For data set B, the average number of identified peptides per sample raised from 97 to 144. Thus, an improvement of about 50% was observed in both cases.

### Estimation of false positives

We validated our method by testing *EIPeptiDi *on data set A, to which 3 LC-MS/MS data from ICAT-labeled HCC-1937 cellular proteins were added. Protein composition in HCC-1937 cells is expected to be totally different from serum protein composition (i.e. the A data set). Thus, any match between *found *peptides from the serum samples and *not found *peptides in the cell lysate (evaluated by *EIPeptiDi*) has to be considered a false positive. False positives were calculated at several tolerance values. The average number of new peptides found in data set A (without considering the cell lysates samples) was evaluated by varying both the mass tolerance and chromatographic retention time tolerance values and are reported in Table 4. Table 5 contains the average number of false positives (in 3 observations) found by running *EIPeptiDi *on the dataset obtained merging the data set A with the three samples composing the data set HCC-1937. Values in the Table 5 refer to the same tolerance values used for Table 4. Let T(i,j) indicate the numbers reported in the Table 4 and let FP(i,j) be the numbers of false positives reported in Table 5. Table 6 reports the false positive rate expressed (in percentage) as the ratio FP(i,j)/T(i,j) at the considered tolerance values. Note that while T(i,j) obviously decreases by narrowing the tolerances, FP(i,j) decreases at an even higher pace, generally causing the false positive rate to decrease constantly by moving down to lower tolerance values. The only exception has been noted for retention time tolerance set at 0.75 min, which, in most cases, caused an increase in the false positive rate. This additional experiment proves that the tolerance values of 30 ppm on mass and 1.5 min on retention time (that are the default tolerances used in our experiments) represent a good compromise between high number of peptides found and a low false positive rate (i.e., 6%). As it can be seen in Table 6, more precise calibration on the mass would improve results even more. For example, 15 ppm mass accuracy or better could be readily achieved by Q-TOF-based MS instrumentation making use of internal calibration or by instrumentation with even higher resolution (e.g. Fourier transform ion cyclotron resonance mass spectrometers, FT ICR, or Orbitrap mass spectrometers). By relying on such mass accuracy, false positives rate is expected to be kept well below 1% (see Table 6), thus in principle allowing peptide matching with no requirements of manual editing, an essential point for undertaking large-scale proteomics experiments. Further experimenting with *EIPeptiDi *may validate this assumption.

**Table 4:** Peptides found in data set A at different tolerances. Average number of peptides found in data set A at different tolerances. The average is evaluated among the seven samples.

	**Mass tolerance: 100 ppm**	**Mass tolerance: 50 ppm**	**Mass tolerance: 30 ppm**	**Mass tolerance: 15 ppm**
**RT tolerance: 5 min**	111.8	107.7	100.9	78.9
**RT tolerance: 3 min**	86.9	86.3	81.9	61.7
**RT tolerance: 1.5 min**	68.5	68.2	66.2	50.4
**RT tolerance: 0.75 min**	20.8	20.2	18.4	13.6

**Table 5:** False positive in data set HCC-1937. Average number of *false positives *at various tolerances found in the data set HCC-1937. The average is evaluated from the three samples composing the data set HCC-1937.

	**Mass tolerance: 100 ppm**	**Mass tolerance: 50 ppm**	**Mass tolerance: 30 ppm**	**Mass tolerance: 15 ppm**
**RT tolerance: 5 min**	24.7	17.0	10.3	3.3
**RT tolerance: 3 min**	13.7	10.7	6.3	1.7
**RT tolerance: 1.5 min**	9.0	7.3	4.0	0.3
**RT tolerance: 0.75 min**	3.3	3.0	1.0	0.3

**Table 6:** False positive rates. False positive rates at various tolerances (values reported in percentages).

	**Mass tolerance: 100 ppm**	**Mass tolerance: 50 ppm**	**Mass tolerance: 30 ppm**	**Mass tolerance: 15 ppm**
**RT tolerance: 5 min**	22.1%	15.8%	10.2%	4.2%
**RT tolerance: 3 min**	15.7%	12.4%	7.7%	2.7%
**RT tolerance: 1.5 min**	13.1%	10.7%	6.0%	0.7%
**RT tolerance: 0.75 min**	16.1%	14.9%	5.4%	2.4%

## Discussion

The technique proposed in this paper presents several advantages over existing software tools available for the data analysis of isotopically labeled samples. First of all, it filters the data, by identifying a quantified peak pair in at least one sample in order for this peak to be considered in further data analysis. In this way, only the most reliable subset of information is exploited. Secondly, the chromatographic retention time alignment step relies exclusively on peaks correctly identified in all samples as calibration points. This way of setting the landmark peaks reduces the risk of peak mismatching to a minimum. Thirdly, MS/MS identifications from several aligned LC-MS/MS data files can be shared, so allowing a results table which contains a considerably higher number of identified peptides and a reduced instance of missing values. The current version of the software has been implemented for ICAT-based platforms. Nevertheless, applications could be expanded in the future to other quantitative MS-based proteomic platforms such as the one based on SILAC [27]. Proteomic approaches using SILAC at the moment rely on the ProQUANT software tool for data analysis, or on the more recently developed AYMUS algorithm [36]. Both tools can perform operations similar to the ones available in *ProICAT*. Although retention time alignment is feasible with ProQUANT, no clustering of MS/MS data is allowed to the user. This dramatically complicates the analysis of sample sets comprising more than only a few samples.

## Conclusion

We designed a framework, called *EIPeptiDi*, that considerably improves *information overlap *in ICAT-based LC-MS/MS studies. The implemented software has been tested and is freely available on line with a user guide and a data set at [37].

## Availability and requirements

Project name: *EIPeptiDi*. The software tool is available at the project home page  and runs on any operating system equipped with a Java Virtual Machine. Instructions on how to run the tool and a database to test it, are published on the project web site.

## Abbreviations

ICAT: isotope-coded affinity tags; LC-MS/MS: liquid chromatography-tandem mass spectrometry; SIC: selected ion chromatogram; PSI: proteomics standards initiative; HUPO: human proteome organisation; TFA: trifluoroacetic acid; FA: formic acid; IDA: information-dependent acquisition.

## Authors' contributions

MC supervised the bioinformatics choices. GC contributed suggestions and supervised the proteomics issues and biological results. MG was responsible for the spectra details intuition and testing the prototype. SG contributed to main paper ideas, algorithms design and data management issues. GT implemented the software tool and defined the architectural choices. PV designed the cross validating framework and the whole software. PV and MG are the principal investigators. All authors read and approved the final manuscript.
